# Health care utilization in the elderly Mexican population: Expenditures and determinants

**DOI:** 10.1186/1471-2458-11-192

**Published:** 2011-03-29

**Authors:** César González-González, Sergio Sánchez-García, Teresa Juárez-Cedillo, Oscar Rosas-Carrasco, Luis M Gutiérrez-Robledo, Carmen García-Peña

**Affiliations:** 1 Instituto de Geriatría. Secretaría de Salud. México City, México (Geriatric Institute. Health Secretariat, Mexico City, Mexico; 2 Unidad de Investigación Epidemiológica y en Servicios de Salud, Área de Envejecimiento. Centro Médico Nacional Siglo XXI; Instituto Mexicano del Seguro Social. México, D.F., México

**Keywords:** Health expenditures, elderly in Mexico, health needs

## Abstract

**Background:**

Worldwide population aging has been considered one of the most important demographic phenomena, and is frequently referred as a determinant of health costs and expenditures. These costs are an effect either of the aging process itself (social) or because of the increase that comes with older age (individual).

**Objective:**

To analyze health expenditures and its determinants in a sample of Mexican population, for three dimensions acute morbidity, ambulatory care and hospitalization focusing on different age groups, particularly the elderly.

**Methods:**

A secondary analysis of the Mexican National Health and Nutrition Survey (ENSANUT), 2006 was conducted. A descriptive analysis was performed to establish a health profile by socio-demographic characteristics. Logistic regression models were estimated to determine the relation between acute morbidity, ambulatory care, hospitalization and age group; to establish the determinants of hospitalization among the population 60 years and older; and to determine hospitalization expenditures by age.

**Results:**

Higher proportion of elderly reporting health problems was found. Average expenditures of hospitalization in households were $240.6 am dlls, whereas in households exclusively with elderly the expenditure was $308.9 am dlls, the highest among the considered age groups. The multivariate analysis showed higher probability of being hospitalized among the elderly, but not for risks for acute morbidity and ambulatory care. Among the elderly, older age, being male or living in a city or in a metro area implied a higher probability of hospitalization during the last year, with chronic diseases playing a key role in hospitalization.

**Conclusions:**

The conditions associated with age, such as chronic diseases, have higher weight than age itself; therefore, they are responsible for the higher expenditures reported. Conclusions point towards a differentiated use and intensity of health services depending on age. The projected increase in hospitalization and health care needs for this group requires immediate attention.

## Background

Recently, worldwide population aging has been considered one of the most important demographic phenomena. It is the product of clear decreases in birth and mortality rates and an increase in life expectancy, which is reflected in the socio-economical progress of countries [1]. The worldwide population aged 60 years and older will surpass from approximately 770 million in 2010 to an estimated one billion in 2020, and 20.0% of these people will be concentrated in developing countries [2]. It is particularly important the increase of the oldest old (people older than 80 years old) and the relatively higher percentage of elderly women, these two phenomenon will be present in almost every country and represent an important economic and social effect [3].

In Mexico, the proportion of elderly population presents the fastest growing in the past 15 years. In accordance with the projections of the National Population Council [4], the current total is 9.4 million people, which represents 8.6% of the total population, and it will increase at an annual rate of 3.9%.

The existent demographic projections also indicate that this process will become more evident in the first decades of this century. The contraction of the population pyramid will become more apparent not only in relative but also in absolute terms: the inertia of the fast increase in the past will become manifest, first in the total number of people in productive age (15-59 years old) and later in the number of people in old age (60 years and over). Adults who are 25 to 59 years old currently represents a total of 48.2 million, and they will continue to increase in numbers until the fourth decade of the present century, when it will reach a maximum of 56.9 million. The amount of elderly people who are 60 years and over will increase even more in the first half of the 21^st ^century [5].

The population in productive age will increase almost 32.0% between 2000 and 2020, only 1.8% until 2035; and there will be a decrease of 6.8% in 2050. On the other hand, the proportion 60 years and over of the total it's growing: this subset of the population will increase from 7.6% in 2005 to 12.1% in 2020, 20.0% in 2035 and 27.7% in 2050. Moreover, the total population will increase in 17.9 million between 2005 to the end of 2050, meanwhile the elderly population will increase in 21.6 million (90.0%) in the same period [6].

The aging process will have important consequences in terms of health. Although it is already known that aging is not necessarily associated with diseases, the cumulative effect of multiple exposures and psychological, physical and social conditions, that are frequently unfavorable, increase the risk of health problems in the elderly [7].

For that reason, the aging process is recurrently referred to as a determinant for the evolution of health costs and expenditures, these costs are either an effect of the aging process itself or a result of the increase that comes with older age. Obviously, if we analyze costs on the individual level, the health costs increase as a function of age [8,9]. Therefore, it may be suggested that the increase in the elderly population will automatically result in an increase in total health costs. However, there is a controversy about this, Mc Grail et al [10] affirmed that the costs for acute care increased with age, but the proximity to death was the important factor. Dormont and Huber [11] reported that in Netherlands, the changes in clinical practice and the new technologies were the causes of the increase in costs. Polder et al [12] revealed that the average cost for younger people are higher than for those who died at advanced ages; however, the growth rate for the per capita costs increase with age, especially when acute care is needed [8].

In developing countries like Mexico, there is no information to confirm or deny this association. It is known that the Mexican elderly have a higher number of health needs, and the utilization rates for health services have increased in people who are 60 years and over [13], but it is not possible to affirm that the costs have increased due to the increase in the number of elderly people.

Therefore, this work aims to analyze this problem further through three dimensions: acute morbidity, ambulatory services use and hospitalization. We will examine the household expenditures associated with these three dimensions according to family structure, living arrangements and age groups. The goal is to verify the assumption that the elderly population uses more intense and frequently the health services than other age groups, because they are significantly more likely to experience health problems, and to asses if the annual average expenditure for hospitalization among the elderly is the highest.

## Methods

A secondary analysis of public access database [14] related to the Mexican National Health and Nutrition Survey (ENSANUT), 2006 was performed. The survey includes information related to the health and nutrition of the Mexican population, as well as, the evolution of the quality and response of health services, the health-related policies and programs that affect population and the healthcare expenditures shouldered by Mexican households. The population included in the ENSANUT 2006 was 48,600 households selected in accordance to a probabilistic, multistage and stratified sampling by conglomerates, with representativeness reflective on the national, regional and state levels [14,15]. For each of the basic geo-statistics areas (AGEB) defined by the National Institute of Statistics and Geography ("Instituto Nacional de Estadística y Geografía") (INEGI), households were selected as blocks of houses with proportional probabilities in its number of houses according to a systematic sampling at random. In each household and when the composition allowed, simple random sampling was used to select an adult (19 years and over), a teenager (10-19 years old), a child (0 to 9 years old) and a health care services user (household members who looked for or received care within the six months prior to the date of the survey for either a disease, lesion, accident, prevention or rehabilitation). The local eligible individuals were survey-adjusted to the correspondent questionnaire (Individual User, Individual Children, Teenagers or Adults), additionally to the household characteristics questionnaire; this resulted in a sampling size of 206,700 records that represented 103,013,985 inhabitants in Mexico in 2006. The household questionnaire was answered by a key informant, presumably the person that better known the household members, and could respond accurately about their characteristics and conditions.

For the purpose of the present analysis, the following variables were considered: a) acute morbidity, defined as a health problem reported from the beginning within 15 days prior to the survey, such as infection of the respiratory tract, acute diarrhea, cephalalgia, allergies and urinary tract infection; b) seeking and granting ambulatory care during the last month; and c) hospitalization during the last year. For hospitalization expenditures, we only included those households with one hospitalized person, in such a way the reported expenditures could be imputed to that person and identify the age group to which they belong.

The following explanatory variables were considered: age, analyzed in four age groups: children between 0 to 9 years old, teenagers between 10 to 19 years old, adults between 20 to 59 years old and adults 60 years and over; gender; insurance, defined as the right of the individual and families to have social security, which includes healthcare among other economical and social benefits; disability, defined as the reported difficulty of an individual to move, walk or move arms, the loss of visual, audio or language acuteness, and/or the presence of mental disability; presence of a chronic disease: hypertension, diabetes, myocardial infarction, kidney disease, stroke and cancer; self report health status which was defined on a scale of six options from very good to very bad; the total number of persons in the household; type of household, which was divided into three categories: "only elderly," "elderly with other people" and "without elderly"; and the locality size of residence, divided into three categories: "metro area", "urban surroundings" and "rural". For hospitalization, we also considered information regarding to hospitalization expenditures, the total amount of money paid by the household members for hospitalization during the last year; however, these spending did not include other costs such as transportation, food and indirect costs.

## Data Analysis

In the first stage, a descriptive analysis was performed for all records (n = 206,700). This analysis established the proportions of the acute morbidity, ambulatory care and hospitalization variables by sex, age group, living arrangements, health insurance, self-report health condition, disability, chronic diseases and household size. Statistical significance was examined using t and χ^2 ^tests as appropriate. If differences remain significant after controlling for demographic and health characteristics, further analysis of the data was conducted. Three logistic regression models were perform to: a) determine whether the relation between acute morbidity, ambulatory care, hospitalization and age were maintained; for these models, 205,138 records were used; b) establish the determinants of hospitalization among the population 60 years and over; 7,618 records were used; and finally, c) determine whether the hospitalization costs varied by age. For this analysis, we included households that had only one person hospitalized in the last year and that had complete information on hospitalization expenditures; therefore, 4,910 records were used.

## Results

A total of 206,700 individuals were included using the population sampling method and were in accordance with the factors of expansion of the ENSANUT 2006; they were representative of the 103,013,985 individuals in all Mexico. We found that 19.7% of the individuals were under 10 years old (50.9% males and 49.1% females), 22.2% were between 10 and 19 years old (50.5% males and 49.5% females), 48.3% were between 20 and 59 years old (46.1% males and 53.9% females) and 9.8% were 60 years and older (45.9% males and 54.1% females).

Regarding to have health insurance, the elderly presented the higher percentage with 59.2%, whereas those under 10 years old had the lowest percentage with 43.2%. In households with at least one individual under 10 years old, the average household size was 5.8 individuals; households with at least one elderly person had the lowest average household size with 3.9 individuals. This percentage was related to the fact that 7.2% of those above 60 years old live alone, whereas around 1.0% of those in other age groups live alone. When asked, "How do you consider your current health status?" the key informant answer that 1.4% of individuals under 10 years old, 1.3% of the teenagers, 3.8% of the adults and 13.5% of the elderly considered bad or very bad their health condition (Table 1).

**Table 1:** Characteristics of the total population by age group, Mexico 2006

					
**Characteristics**	**Age Groups (years)**				
	
	**Under 10**	**10 to 19**	**20 to 59**	**60+**	**Total**

**N**	42,149	47,875	97,297	19,379	206,700
Estimated population	20,216,780	22,874,888	49,786,891	10,135,426	103,013,985
Percentage of the total	19.6	22.2	48.3	9.8	100.0
					
**Gender**					
Men	50.9	50.5	46.1	45.9	48.0
Women	49.2	49.5	53.9	54.1	52.0
					
**With health insurance**	43.2	43.6	48.0	59.2	47.2
					
**Where there is at least one... the household size is...**					
Household size	5.8	5.8	5.1	3.9	5.3
					
**Health status**					
Very good and Good	80.4	80.5	66.9	44.4	70.4
Regular	18.2	18.2	29.3	42.1	25.9
Very bad and Bad	1.4	1.3	3.8	13.5	3.8

Table 2 shows the percentages of acute morbidity, ambulatory care and hospitalization according to the characteristics of the sampled population for the study.

**Table 2:** Percentage of population that reports acute morbidity, ambulatory care and hospitalization by social demographic characteristics, Mexico 2006

Variables	Percentages of		
	**Acute Morbility**^1^	**Ambulatory care**^2^	**Hospitalized**^3^

**Gender**			
Men	10.3	9.9	2.5
Women	11.6	7.3	4.3
**Age Group**			
Under 10 years old	14.9	11.6	2.0
10 to 19	7.8	5.0	1.5
20 to 59	11.7	7.8	4.3
60+	18.7	15.9	6.8
**Household type**			
Only elderly	21.6	17.6	6.3
elderly with others	11.7	9.2	4.0
Without elderly	12.0	8.2	3.2
**Health Insurance**			
No	10.0	6.7	2.5
Yes	12.0	10.6	4.3
**Health Condition**			
Very good, Good or Regular	10.0	8.0	3.1
Bad or Very bad	38.7	26.4	13.1
**Disability**			
Without disability	10.6	8.3	3.2
With disability	23.9	20.2	12.8
**Chronic disease**			
**Without **Chronic disease	9.8	7.6	2.8
**With **Chronic disease	26.2	23.2	12.1
**Household size**			
1	21.9	18.4	6.5
2	16.5	13.9	5.5
3 to 5	11.7	9.2	3.5
6 to 10	9.0	7.0	3.0
11+	6.7	4.5	2.5
**Locality size**			
Rural	11.2	7.6	2.5
Urban Surroundings	11.6	8.6	3.6
City or metro area	12.9	9.3	3.9

Our analysis of the data from ENSANUT, 2006

1 In the last weeks, have you had any type of health problem?

2 In the last weeks, have you sought or received ambulatory care?

3 During the last year, has someone from this household been hospitalized?

The Chi-square and t-tests were used to review whether there were significant differences in the means or percentages of the variables based on each case. All were significant at 95%.

In the three dimensions analyzed, the elderly presents a higher percentage than any other population age groups. We found that 18.7% of the elderly reported a health problem within the last two weeks, whereas 14.9% of those under 10 years old, 11.7% of those between 20 and 59 years old and 7.8% of teenagers reported a health problem in the same period. With regard to ambulatory care, 15.9% of the elderly sought or received ambulatory care during the last two weeks, while 11.6% of children, 7.8% of adults and 5.0% of the teenagers also did so. For hospitalization, the results show that 6.8% of the elderly spent at least one night hospitalized in the last year, whereas the percentages for adults, children and teenagers were 4.3%, 2.0%, and 1.5%, respectively (Table 2).

Women presented a higher percentage of acute morbidity and hospitalization, while men reported the highest percentage of ambulatory care. Those individuals with health insurance had the highest percentage of the population with health problems, ambulatory care and hospitalization compared with uninsured individuals.

In comparison with those who reported very good, good or regular health status, those who reported a bad health status constituted a higher percentage of population in the three dimensions examined, and the differences between these two groups were wide. Similarly, those with disability or chronic disease also constituted the highest percentage in the three dimensions examined. When we analyzed living arrangements, households with only elderly persons had higher percentages for acute morbidity (21.6%), ambulatory care (17.6%) and hospitalization (6.3%) compared with the extended or complex households (households with elderly and other members or households without elderly). With regard to the locality size, the larger the size the higher the percentage of the population that reported a health problem, needed ambulatory care and were hospitalized (Table 2). The bivariate analysis between the acute morbidity, ambulatory care and hospitalization variables established that there were statistically significant differences between the dependent variables and the variables gender, age group, living arrangements, health insurance, self reported health status, disability, chronic diseases and locality size (p < 0.05) (Table 2).

For hospitalization expenditures averages during the last year in Mexican households, we found that the average expenditure was $3,044 pesos (240.6 am dollars) In August 2010, the exchange rate was 12.65 Mexican pesos per 1 U.S. dollar. By gender, the expenditure was higher for men $3,741 pesos (295.7 am dollars); in those who were 60 years and older, the expenditure was $3,907 pesos (308.9 am dollars), the highest expenditure among the considered age groups; by household type, those with older persons and another individual living with them and that had at least one individual hospitalized, the annual average expenditure was $3,704 pesos (292.8 am dollars); those who did not have social health insurance spent $4,261 pesos (336.8 am dollars), almost twice the money spent by those with health insurance. For those who self reported bad health status, those who had disabilities or those who had been diagnosed with chronic diseases, the average hospitalization expenditures were $3,570 (282.2 am dollars), 3,208 (253.6 am dollars) and 3,371 pesos (266.5 am dollars), respectively (data not shown).

### Multivariate Analysis

Among the general population (205,138 records), the results of the regression models suggested that after controlling for demographic and health variables, the elderly presented a higher probability of being hospitalized than children between 0 to 9 years old, with an odds ratio of 2.53 (CI 95%, 2.24-2.86). The risks for acute morbidity and ambulatory care were lower for the elderly than for the children, with odds ratios of 0.81 (CI 95%, 0.75-0.87) and 0.87 (CI 95%, 0.81-0.94), respectively. The results reported in the bivariate analysis do not match with those obtained with the regressions.

Among women, population with health insurance, those who considered their health status to be bad or very bad, those who had at least one chronic disease and those who had some type of disability, the probability of hospitalization, acute morbidity or requiring ambulatory care is higher than for those in the reference category. According to household structure, households with only elderly people had the lower probability in each three dimensions, and the reference category was households without elderly people. Living in an urban area increased the probability of hospitalization, seeking ambulatory care, and having health problems (Table 3).

**Table 3:** Logistic regression models for acute morbidity, emergency care and hospitalization, Mexico 2006 (n = 204,997)

Covariates	Acute Morbidity		Ambulatory care		Hospitalization	
	
	Odds Ratio	Confidence Interval	Odds Ratio	Confidence Interval	Odds Ratio	Confidence Interval
**Age Group**						
*Children*	*1.000*		*1.000*		*1.000*	
Teenagers	0.469***	(0.448-0.491)	0.388***	(0.369-0.409)	0.761***	(0.688-0.843)
Adults	0.559***	(0.539-0.580)	0.485***	(0.466-0.505)	1.806***	(1.670-1.952)
Elderly	0.808***	(0.754-0.866)	0.870***	(0.807-0.938)	2.532***	(2.239-2.863)
						
Men	0.913***	(0.888-0.940)	0.735***	(0.712-0.759)	0.614***	(0.584-0.646)
						
Eligible for SS	1.128***	(1.096-1.161)	1.516***	(1.467-1.566)	1.555***	(1.478-1.636)
						
With disability	1.418***	(1.318-1.525)	1.513***	(1.400-1.634)	2.459***	(2.236-2.705)
						
With chronic disease	2.670***	(2.557-2.789)	2.992***	(2.858-3.133)	2.914***	(2.741-3.098)
						
Bad health condition	4.490***	(4.257-4.737)	2.929***	(2.758-3.111)	2.653***	(2.448-2.875)
**Household**						
*Only Elderly*	*1.000*		*1.000*		*1.000*	
Elderly with others	1.006	(0.921-1.098)	1.052	(0.961-1.152)	1.126	(0.984-1.289)
Without elderly	1.244***	(1.129-1.371)	1.295***	(1.170-1.435)	1.541***	(1.321-1.797)
						
Household total	0.922***	(0.915-0.929)	0.914***	(0.906-0.922)	0.998	(0.986-1.010)
**Locality size**						
*Rural*	*1.000*		*1.000*		*1.000*	
Urban surroundings	1.033	(0.994-1.074)	1.085***	(1.039-1.132)	1.207***	(1.126-1.293)
City or metro area	1.061**	(1.024-1.099)	1.067**	(1.026-1.110)	1.260***	(1.183-1.342)

* p < 0.05, ** p < 0.01, *** p < 0.001

In regard to hospitalization expenditures, results from regression models suggest that among hospitalized population (4,910 records), individuals 60 years and older spent on average $2,115 pesos (167.2 am dollars) (CI 95%, 768.22-3461.17) more than the reference group (children from 0 to 9 years), and individuals between 20 to 59 years old spent on average $921 pesos (72.8 am dollars) (CI 95%, 9.86-1831.61) more, and those between 10 to 19 years old spent on average $462 pesos (36.5 am dollars) (statistically not significant) (CI 95%, -710.68-1634.49) more than the reference group. The participants who had health insurance on average spent $2,500 pesos (197.6 am dollars) less than those who did not have insurance. Household structure had no significant effect on hospitalization expenditures even though the households in which there were older adults living with individuals of another age group had a higher hospitalization cost (Table 4).

**Table 4:** Linear regression model on hospitalization expenditures (Mexican pesos), Mexico 2006 (n = 4,910)

Covariates	Hospitalization Costs	
	
	Coefficient.	Coefficient Interval
		
**Age Group**		
*Children*	*1.000*	
Teenager	461.925	(-710.638-1634.488)
Adults	920.737*	(9.865-1831.609)
Elderly	2114.697**	(768.222-3461.172)
		
Men	870.999**	(307.281-1434.717)
		
Eligible for SS	-2499.838***	(-3052.617--1947.058)
		
With disability	-181.687	(-1116.146-752.771)
		
With chronic disease	225.309	(-391.800-842.418)
		
With bad heath condition	275.750	(-539.335-1090.834)
**Household**		
*Only Elderly*	*0.000*	
Elderly with others	866.215	(-545.476-2277.906)
Without elderly	494.562	(-1129.228-2118.351)
		
Household total	-89.218	(-224.501-46.064)
**Area size**		
*Rural*	*0.000*	
Urban surroundings	-473.168	(-1218.720-272.384)
City or metro area	75.090	(-608.654-758.833)
		
Constant	3064.869**	(1168.017-4961.721)

* p < 0.05, ** p < 0.01, *** p < 0.001

Results suggested that population 60 years and over spent more money for hospitalizations; therefore, we analyzed this age group with variables that had an effect on being hospitalized during the last year (7,618 records). Older age, being male or living in a city or in a metro area implied a higher probability of hospitalization during the last year. Among the chronic diseases, those with cancer has the higher probability of been hospitalized (OR 2.95, CI 95% 1.71-5.09), followed by those with Myocardial Infarction (OR = 2.47, CI 95% 1.95-3.14) and those with stroke (OR = 2.13, CI 95% 1.27-3.58). The probability of being hospitalized due to an accident was 1.5 times higher than for those who did not have an accident (Table 5).

**Table 5:** Logistic regression model on hospitalization determinants in the population 60 years old or older, Mexico 2006

Covariates	Odds Ratio	CI 95%
		
Age	1.018**	(1.006-1.030)
		
Men	1.268*	(1.034-1.555)
**Locality size**		
*Rural*	*1.000*	
Urban surroundings	1.044	(0.801-1.359)
City or metro area	1.327*	(1.033-1.706)
		
Diabetes	1.508***	(1.203-1.891)
		
Hypertension	1.443***	(1.183-1.761)
		
Myocardial infarction	2.472***	(1.946-3.140)
		
Kidney disease	1.421**	(1.132-1.783)
		
Cerebrovascular accident	2.132**	(1.270-3.579)
		
Cancer	2.951***	(1.711-5.088)
		
Accident	2.517***	(1.899-3.337)
		
Eligible for SS	1.951***	(1.563-2.435)
**Education**		
*Without education*	*1.000*	
Elementary incomplete	0.878	(0.706-1.091)
Elementary complete	0.582**	(0.393-0.862)
Beyond elementary school	0.805	(0.471-1.375)
		
With partner	1.037	(0.843-1.275)
		
Bad health status	2.622***	(2.109-3.260)

n = 7,618

* p < 0.05, ** p < 0.01, *** p < 0.001

**Figure 1:**
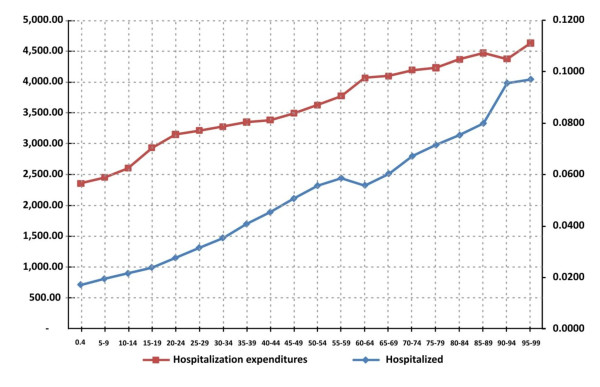
Probability of being hospitalized and average hospitalization expenditures (Mexican pesos) by quinquennial groups, Mexico, 2006

Finally, Figure 1 shows the probability of being hospitalized and the household hospitalization expenditures by single age. Higher age is associated with higher probability of hospitalization, the probability of being hospitalized are less than 2.0% for those between 0 to 4 years old and approximately 10.0% for those between 95 to 99 years old. The hospitalization expenditures were higher for those in older age; the minimum expenditure per household was approximately $2,400 (189.7 am dollars) and the maximum $4,700 pesos (371.5 am dollars).

## Discussion

This report analyzed secondary data from the ENSANUT 2006 to explore differences by age group. Our main findings suggest that the population 60 years and older reported a higher percentage of acute morbidity, sought or received ambulatory care and hospitalization. This research was base on the premise that the needs and costs of medical care are different by age group. The study highlighted that, as part of demographic dynamics, there will be a progressive and accelerated population aging process as well as a reconfiguration of the age structure. Therefore, it is necessary to study further the health needs, the expenditures and determinants of hospitalization among Mexican household members.

Elsewhere, there has been an increasing interest in elucidating whether the aging process represents a burden to health care services. Berenstein CK and Wajnman S [16] analyzed the health care costs in two cities of Brazil, they report that only 13% of health costs may be allocated to changes in the age structure, and a great proportion (72%) was due to price effects. A recent article of Werblow et al. [17] analyzed whether there were biases of interpretation due to the aging effect in health care costs; they used data from 91,327 people from Zurich and Geneva and concluded that at an aggregated level, age had an insignificant effect on individual healthcare costs but, healthcare costs were closely related to the proximity of death.

Changes that are present with Mexican population aging forces an analysis of the probable consequences that these changes could represent for the society and for the health system. In Mexico, there are currently 5.8 million people over 60 years old, and by 2050 it is expected that the population increases to 25.9 million, which means quadrupling the aged population in less than fifty years [4]. There are limited analyses on what this increase will represent in terms of needs and requirements, this research aimed simultaneously to fill that gap and to inform about the challenges that the increase in the elderly population will bring. The current estimates for health care needs, the incurred household's expenditures and the factors intervening in the hospitalization represent an advance and a platform for future research.

The results from descriptive data: higher percent of elderly population with acute morbidity, sought and received ambulatory care and higher hospitalization, were not in compliance with the regression models and were opposite to the expected results. To verify these findings, we developed regression models that included age groups as the only explanatory variable, and the results suggested the same result found in the bivariate analysis: higher acute morbidity, higher ambulatory care and higher hospitalization occurred among the elderly than among children and the other age groups. However, once the rest of the explanatory variables were included, the age effect changed for acute morbidity and ambulatory care of the elderly. Therefore, regression coefficients indicated that the age of the elderly group by itself was not a factor in presenting health problems in these two dimensions. The presence of other factors associated with aging, such as chronic diseases, play a key role, leading us to conclude that the conditions associated with age have more weight than age itself; therefore, they are responsible for the higher costs reported [18]. Then it is possible that, as described by Polder et al. [8] technological and epidemiological changes are the responsible factors for the increases in costs. Obviously, the reported data only include a very limited vision of what the health needs are in the long term, and other aspects of medical practice were not considered in the present analysis and further research is needed[19].

Likewise, the living arrangements did not have the expected direction in analysis. The households without elderly members had higher probabilities in the three dimensions analyzed. Possibly part of the effect was captured with the age groups and locality size. Also, the considered health problems were diverse: the hospitalization dimension included obstetric procedures such as delivery and cesareans. When we made a revision of the hospitalization causes, in 89.4% of households with only elderly members the hospitalization cause was surgery or disease, this percentage decreased to 57.8% in the households without elderly. It is probable that in the households without elderly members the higher probabilities in the three dimensions were related to acute health, obstetric and child health problems. In addition, it is possible to assume that the elderly living alone are in better condition than those who live with other people. In fact, when comparing those who live alone with those who live with other people, we found that the first group presented lower percentages of hospitalization and lower rates of disability and chronic diseases, among other conditions.

In fact, several authors have suggested the association between living arrangements and health. Among the elderly, the decision to live alone is preceded with a good health condition [20]. Montes de Oca and Hebrero [21] with data from the Mexican Health and Aging Study (MHAS) found that individuals with a better health condition were more likely to live alone, maybe because their condition allowed them to have an independent and autonomous life. Also, it has been documented that among the elderly, there is a preference for living alone or with their spouses when health conditions allowed [22].

Data regarding to health insurance shows that population with health insurance had more health problems, needs more ambulatory care and hospitalization compared with uninsured individuals. These differences probably are explained by the access to health care services. Those who had health insurance have the opportunity of being diagnosed, treated and hospitalized. It is worth to mention that the Mexican public health system is characterized by the presence of several vertically integrate insurer/providers serving different parts of the population with little connection between them. Social security institutions covers salaried workers in the formal sector and the Ministry of Health that also provides health services covers around 40% of population uninsured by social security. Private insurance sector remains small [23]

It is necessary to admit that the study had some limitations; this was a secondary analysis of the ENSANUT 2006, we are limited in the detailed analyses that we can perform, the needs of health care and hospitalization causes were very diverse among the age groups, which makes the comparison difficult. Also, it was not possible to integrate other important causes for the elderly population, for example, the ones related to geriatric syndromes in which falls represent one of the main causes for health care needs, high costs and hospitalization; as well as adverse events caused by pharmacological interactions.

The National survey reported here (ENSANUT 2006) is probabilistic; however, we have to admit that likely a "recall period" bias, defined as the period over which respondents are required to remember past events, may be present. In addition, the selective recall in the hospitalization expenditures can lead to remember more accurately some events, especially those for children or adults where the expenditures can increase within the previous year to the interview [24].

Although ENSANUT is representative by State, analysis by region, particularly the borders are not possible. Particularities of these areas such as the constant flux of persons between countries and the specific socioeconomic characteristics should be addressed in further research as an opportunity to understand and reveal some issues about health dynamics, for example: cost, quality, and/or accessibility.

Until now, there have been a lack of studies of this type in Mexico, we have begun to uncover the interaction between aging process and health, and these results show an approximation of these health needs. Our study contributes to the comparability of information among countries with the same demographic dynamics, but also with developed countries. Our research can be a reference for future research especially in the Latin-American and in countries with accelerated aging process. Health care expenditures and their determinants have attracted considerable attention and have been used widely to compare countries. The most direct link between population aging and national health expenditure is due to the fact that per-capita health spending increases with age. The estimated ratio of health spending by age groups, and specifically within the elderly, it's an important issue, since the increase in health expenditures along with the increase in proportion of population aging is observed in almost all countries.

## Conclusions

Conclusions points towards a differentiated uses and intensity of health services depending on age. Among the elderly population, hospitalization is "the topic" to be explored further, particularly once the population of those 60 years and older increases and the health care needs for this population also grow.

## Competing interests

The authors declare that they have no competing interests.

## Authors' contributions

CGG design the study, prepared the database and conduct the analysis. SSG and TJC contribute to the conception of the study, interpreted the data and review the manuscript. ORC and LMGG were involved in the design of the study, the interpretation of the data and the revision of the manuscript. CGP had the original idea, collaborated with the design, the analysis and review critically the manuscript. All gave final approval of the submitted version.

## Pre-publication history

The pre-publication history for this paper can be accessed here:

http://www.biomedcentral.com/1471-2458/11/192/prepub
